# Modelling *Salmonella* Typhi in high-density urban Blantyre neighbourhood, Malawi, using point pattern methods

**DOI:** 10.1038/s41598-024-66436-9

**Published:** 2024-07-26

**Authors:** Jessie J. Khaki, James E. Meiring, Deus Thindwa, Marc Y. R. Henrion, Tikhala M. Jere, Harrison Msuku, The STRATAA Consortium, Amit Aryja, Archana Maharjan, Sabina Dongol, Abhilasha Karkey, Binod Lal Bajracharya, David Banda, Clemens Masesa, Maurice Mbewe, George Mangulenji, Chisomo Msefula, Tonney Nyirenda, Yama F. Mujadidi, Merryn Voysey, Jennifer Hill, Pallavi Gurung, Arifuzzaman Khan, Nirod Chandra Saha, Prasanta Kumar Biswas, Anup Adhikari, Robert S. Heyderman, Melita A. Gordon, Emanuele Giorgi

**Affiliations:** 1 The Centre for Health Informatics, Computing, and Statistics (CHICAS), Lancaster University, Lancaster, UK; 2 Malawi Liverpool-Wellcome (MLW) Trust Programme, Blantyre, Malawi; 3 School of Global and Public Health, Kamuzu University of Health Sciences, Blantyre, Malawi; 4 Department of Infection, Immunity and Cardiovascular Disease, University of Sheffield, Sheffield, UK; 5 Department of Epidemiology of Microbial Diseases, Yale University, New Haven, USA; 6 Department of Clinical Sciences, Liverpool School of Tropical Medicine, Liverpool, UK; 7 Division of Immunity and Infection, Veterinary and Ecological Sciences, University College London, London, UK; 8 Institute of Infection, Veterinary and Ecological Sciences, University of Liverpool, Liverpool, UK; 9 Oxford University Clinical Research Unit, Patan Academy of Health Sciences, Kathmandu, Nepal; 10 Wasa Pasa Polyclinics Private, Lalitpur, Nepal; 11 Kamuzu University of Health Sciences, Blantyre, Malawi; 12 Oxford Vaccine Group, Department of Paediatrics, University of Oxford, Oxford, UK; 13 NIHR Oxford Biomedical Research Centre, Oxford, UK; 14 Patan Academy of Health Sciences, Kathmandu, Nepal; 15 International Centre for Diarrhoeal Disease Research, Dhaka, Bangladesh; 16 Nepal Family Development Foundation, Lalitpur, Nepal

**Keywords:** Statistics, Risk factors

## Abstract

*Salmonella* Typhi is a human-restricted pathogen that is transmitted by the faecal–oral route and causative organism of typhoid fever. Using health facility data from 2016 to 2020, this study focuses on modelling the spatial variation in typhoid risk in Ndirande township in Blantyre. To pursue this objective, we developed a marked inhomogeneous Poisson process model that allows us to incorporate both individual-level and environmental risk factors. The results from our analysis indicate that typhoid cases are spatially clustered, with the incidence decreasing by 54% for a unit increase in the water, sanitation, and hygiene (WASH) score. Typhoid intensity was also higher in children aged below 18 years than in adults. However, our results did not show evidence of a strong temporal variation in typhoid incidence. We also discuss the inferential benefits of using point pattern models to characterise the spatial variation in typhoid risk and outline possible extensions of the proposed modelling framework.

## Introduction

*Salmonella enterica* serovars Typhi (*S.* Typhi) is a human-restricted pathogen that is transmitted by the faecal-oral route and the causative organism of typhoid fever. *S.* Typhi is estimated to cause more than 10.9 million cases each year, with about 116,000 of the cases resulting in death^1,2^. Whilst the global incidence of typhoid is estimated at 293 cases per 100,000 person-years, the highest burden of typhoid is reported to be in resource-constrained settings, particularly in sub-Saharan Africa and South Asia^2,3^. A meta-analysis in 2017 estimated a typhoid incidence of 149 cases per 100,000 person-years in southern sub-Saharan Africa, whilst South Asia was estimated to have a typhoid incidence of 204 cases per 100,000 person-years^2^.

Typhoid is primarily transmitted when a healthy person comes into contact with stool-contaminated food or water^3–5^. Inadequate access to clean water and sanitation are thus two of the main risk factors associated with typhoid^6^. One study has indeed shown that, in Malawi, typhoid risk is highly affected by the type of water that a household uses for cooking and cleaning^5^. Elevation also plays an important role in the risk of typhoid infection. A study in Kenya showed that individuals, particularly children, living in low-elevation areas were twice more likely to contract typhoid than people living at higher elevations^4^. This can be explained by the accumulation of faecal waste in low-elevation areas due to the downstream flow of contaminated water^4^. Recent studies^3,7^ have also reported that rainy seasons are associated with an increased risk of typhoid, suggesting that the occurrence of typhoid follows a seasonal pattern with variations dependent on the climatic and environmental conditions of the region. On the other hand, heavy-intensity rainfall is shown to have a negative association with typhoid incidence as the high-intensity rainfall may wash away faecal substances^7^.

The risk of typhoid also varies across different groups of age and gender. Several studies have shown that the burden of typhoid is highest among children between 5 and 19 years, an age group typically identified as school-going children^1^. A study in Blantyre, Malawi, showed that the highest typhoid-attributable risk percentage among the children in the study arose from spending a day in a daycare or school^5^. This result is in agreement with the results from another study where the incidence of typhoid was highest among children aged 5–9 years, followed by those aged between 2 and 4 years^8^. Evidence of the effect of gender on typhoid is, on the other hand, contradictory. While other studies have shown that both occurrences of typhoid and mortality due to typhoid are higher among males^1^, others have reported a higher occurrence of typhoid among females^9^.

Typhoid is monitored using passive or enhanced surveillance methods depending on a country’s level of endemicity and public health objectives. The World Health Organisation (WHO) recommends that endemic countries such as Malawi should have, as a minimum, laboratory and facility-based surveillance^10^. The surveillance can be carried out through passive reporting of results from the laboratory, the establishment of a surveillance system, or active review of laboratory records to find patients whose results meet the criteria for a confirmed typhoid case^10^. The WHO, additionally, recommends surveillance through population-based studies to estimate the population-based incidence of a country and generate information for programmatic interventions^10^. In this study, we used data collected from a passive surveillance study in Malawi^6,11,12^.

In Malawi since 1998, blood cultures have been routinely collected from febrile patients at Queen Elizabeth Central Hospital (QECH) in Blantyre^13^. A study showed that an average of 14 cases per year were recorded between 1998 and 2010 at QECH^14^. The same study also reported a rapid increase in typhoid cases starting from 2011, with a peak observed in 2014 at 782 cases^14^. The outbreak of typhoid in both Malawi and other African countries is due to a multidrug-resistant (MDR) typhoid strain to ampicillin, chloramphenicol, and cotrimoxazole that originated in Asia^14,15^. The escalating issue of antimicrobial resistance (AMR) is a threat to global health as current drug AMR trends may hinder efforts to control typhoid through antibiotic treatment and lead to an increase in the risk of typhoid worldwide^16^.

Understanding the spatial variation in the risk of typhoid can help to identify disease hotspots and develop more targeted control interventions. Spatial and spatio-temporal statistics can thus play a critical role by utilising information across time and space and making the best use of data from constrained resource settings. Among previous typhoid research, some studies have used a quasi-Poisson generalised linear model and an over-dispersed Poisson generalised linear model to assess the relationships between typhoid and climatic variables, such as temperature and rainfall^7,17^. Another previous study in Blantyre, Malawi, used geostatistical methods to model and map the inhomogeneous distribution of typhoid genomic data^18^. Similarly, a study from Ghana has shown that typhoid incidence at the district level exhibits spatial and temporal patterns and modelled that using a negative binomial autoregressive moving average model^19^. Another study in Uganda used a spatial scan statistic for incidence to identify hotposts and a standard Poisson model with no overdispersion to investigate spatio-temporal trends of typhoid^20^. One of the main drawbacks of spatial scan statistics is the inability to correctly identify non-circular or irregularly shaped clusters^21^. Our work builds on the current literature by developing a spatially explicit statistical model for point pattern process typhoid data.

The focus of this paper is to develop a spatial point pattern model to assess the effect of environmental and individual risk factors on typhoid fever, using health facility data. To the best of our knowledge, this is the first study that uses spatial point pattern models for the analysis of geo-located typhoid cases. This work, therefore, extends prior research on geostatistical modelling of typhoid genomic data in Blantyre, Malawi, by modelling geo-located households using both individual-level and spatial covariates in the modelling^18^. The specific objectives of the study were as follows:

- To investigate the association between spatial and temporal covariates with the occurrence of typhoid in Ndirande township after adjusting for individual-level markers, namely age and gender and;
- To investigate spatial and temporal trends of typhoid in Ndirande township.

## Methods

### Study site

The study was conducted in Ndirande township in Blantyre city in Malawi between October 2016 and February 2020. Ndirande, which had a population of about 100,000 people in 2018, spans an area of approximately 6.7 km^2^ and is serviced by one government health clinic^6^. Blantyre city, which is in the southern part of Malawi, lies $35^{\circ }$ east of Greenwich Meridian and $15^{\circ }$ south of the Equator. Blantyre city was selected for the study because of the well-known high burden of typhoid fever and the research capacity to carry out complex studies^6^.

Malawi has two main climate seasons: the rainy and dry seasons. The rainy season can be further distinguished between the early rain (November–February) and the late rain (March–April) seasons^7^. Similarly, the dry season can also be distinguished into the cool dry (May–August) and the hot dry (September–October) seasons^7^. A recent study protocol reported that the number of typhoid cases per month in Ndirande township in Blantyre district in Malawi increased in the months of December through February, which corresponds to the rainy season in Malawi^6^. Ndirande exhibits a variation in elevation, ranging from 970 to 1200 meters, with a median elevation of 1118 meters. Total precipitation also varied from 819 millimeters (mm) to 1602 mm from 2016 to 2019. The variation in total precipitation across Ndirande was, however, minimal with the maximum difference being 209 mm each year. In this study, we included season as a temporal covariate in our modelling.

### Data

#### Passive surveillance study of the STRAATA project

The Strategic Typhoid Alliance across Africa and Asia (STRATAA) study was carried out in Bangladesh, Nepal and Malawi with the aim of measuring the burden of typhoid in these three sites^6^. In Malawi, the STRATAA study was carried out by the Malawi-Wellcome-Liverpool Clinical Research Programme at the government-run Ndirande health clinic, which is the largest clinic in Ndirande township. In this paper, our focus is on the passive surveillance sub-study of the STRAATA project.

In the passive surveillance study, patients presenting with a history of fever for at least 2 days or a patient presenting with a temperature of at least 38.0 °C at the Ndirande health clinic were approached with the intention of enrolling them into the study^6,12^. Passive surveillance was, additionally, performed at Queen Elizabeth Central Hospital (QECH) for patients from Ndirande who presented to the Accident and Emergency Treatment Centre (AETC) or were admitted to the wards^12^. A blood culture was collected from the patients who consented to be enrolled in the study. A total of 161 typhoid cases were recorded at Ndirande health clinic in a passive surveillance study between October 2016 and February 2020. The gender and age of the study participants were collected as part of the routine data collected in the study. However, 1 case did not have a date of collection and was therefore excluded from the analysis. Handheld Global Positioning Systems (GPS) devices were used to collect the locations (latitude and longitude) of the households of the typhoid cases.

Two marks, namely the gender (male or female) and age in years of a typhoid case were included in our model. Age was categorised into 3 levels (0–5, 6–17 and 18+ years) given previous studies on the association between typhoid and several age groups^5,8^.

#### Population data

The STRATAA study also carried out household and individual-level population censuses in 2018. The population census, which enumerated 102,242 individuals, was used as an offset in the model.

### Spatial covariates

Covariate selection was informed by previous research on the associations between typhoid and environmental covariates^4,5,17,22,23^. For this study, we restricted our attention to those covariates that are available at a spatial resolution of 100 m^2^ for Ndirande. Hence, our spatial covariates are: distance to Ndirande health clinic in meters, elevation (in meters) and a Water, Sanitation and Hygiene (WASH) score.

The distance to the health clinic raster was derived by calculating the Euclidean distances from each location within Ndirande township to the health clinic. The elevation raster file was downloaded from the WorldPop website^24^. The raster was cropped to a 100 m^2^ Ndirande grid.

A water, sanitation, and hygiene (WASH) survey was carried out in 14,136 households in Ndirande township in 2018 as part of the STRATAA study. The WASH variables were self-reported in the questionnaire. A WASH score was derived using principal components analysis (PCA), and a linear geostatistical model was used to interpolate the WASH score over the grid. Further details on the spatial covariates, including how the WASH score was derived, are supplied in the supplementary material.

### Modelling of reported typhoid fever cases using point-pattern models

We develop an inhomogeneous spatial marked point process model that allows us to incorporate both spatial covariates and individual-level covariates as marks^25^. Let *i* denote the subscript for gender, with $i=1$ corresponding to “male” and $i=2$ to “female”. We then use *j* to denote the subscript that identifies a specific age group, $j=1$ representing individuals between 0 and 5 years, $j=2$ between 6 and 17 years, and $j=3$ for those above 17 years. Our outcome variable corresponds to the locations of the reported diagnosed cases *x* that fall in *A*, representing the area encompassed by the boundaries of Ndirande township. It, therefore, follows that $n_{ij}$ corresponds to the number of typhoid cases in a specific age-gender combination. By setting age and gender as marks, we model the cases reported within each age-gender subgroup as independent inhomogeneous Poisson processes. More specifically, we model the intensity of the subgroup for gender *i* and age *j* as $\lambda _{ij}\left( {x}\right) = \exp \left( \alpha _{i} + \gamma _{j} + d\left( {x} \right) ^{\prime }\beta + \log {m_{ij}(x)} \right) .$ In the equation for the intensity, we use $\alpha _{i}$ to account for the gender effects and $\gamma _{j}$ to account for differences across age groups. The vector $d\left( {x} \right)$ denotes a linear combination of spatial covariates: distance, measured in meters, to Ndirande health clinic ($\beta _1$); elevation, in meters ($\beta _2$); and the WASH score ($\beta _3$). Finally, ${m_{ij}(x)}$ is an offset corresponding to the population for an individual with gender *i* and age *j* at location *x*.

We denote the vector of unknown parameters with $\theta$, which consists of intercepts quantifying the gender effects ($\alpha _i$, for $i=1,2$) and age effects ($\gamma _j$, for $j=1,2,3$) and the regression coefficients $\beta$. The likelihood function for $\theta$ is then given by

1 $$\begin{aligned} L(\theta ) = \sum _{i=1}^{2} \sum _{j=1}^{3} L_{ij}(\theta ) \end{aligned}$$

where

2 $$\begin{aligned} L_{ij}(\theta )=\sum _{k=1}^{n_{ij}} \log \lambda _{ij} \left( {x}_{k} \right) - \int _{A} \lambda _{ij}\left( {x} \right) d {x} \end{aligned}$$

We use a quadrature procedure to approximate the integral in (2) based on a 100m by 100m regular grid of the study area denoted as *A*^26^. To obtain confidence intervals for the parameters $\theta$, we use parametric bootstrap^27^ based on the following iterative steps.

1. Simulate N= 10,000 samples from the fitted point process model with mean:
  3 $$\begin{aligned} \lambda _{ij}\left( {x}\right) = \exp \left( \alpha _{i} + \gamma _{j} + d\left( {x} \right) ^{\prime }\beta + \log {m_{ij}(x)} \right) \end{aligned}$$
2. Fit the model to the N bootstrap realisations simulated in step (1).
3. Store parameter estimates from each of the fitted models.
4. Use the percentile method to get a 95% confidence interval from the estimates stored in step (3).

We fitted both a spatial model (2) and spatio-temporal model (equation 3 in the supplementary information) to our data. We tested for temporal trends in the data by comparing the purely spatial model and model with temporal covariates using a likelihood ratio test under the null hypothesis that the spatial model should be used to fit the data.

We computed predicted incidence rates for each combination of marks (age and gender) while adjusting for the spatial covariates and population as defined in the intensity equation above $\left( \lambda _{ij}\left( {x}\right) = \exp \left( \alpha _{i} + \gamma _{j} + d\left( {x} \right) ^{\prime }\beta + \log {m_{ij}(x)} \right) \right)$. In addition to plotting the age and gender predicted incidence rates on the 100m by 100m regular grid, we also estimated the area-wide incidence for Ndirande, defined as

4 $$\begin{aligned} \frac{\int _{A} \lambda _{ij}(x) dx}{ \int _{A} m_{ij} (x) dx}. \end{aligned}$$

The integrals in Eq. 4 were approximated using a regular grid with a spatial resolution of 100m by 100m.

#### Model validation

To validate the compatibility of the spatial point pattern model presented in the previous section with the data, we develop a simulation procedure based on the K-function, which is expressed as^28^

5 $$\begin{aligned} {\widehat{K}}(r)=\frac{1}{D|W|} \sum _{h} \sum _{k \ne h} \frac{I\left\{ || x_{k}-x_{h}|| \le r\right\} }{\hat{\lambda }\left( x_{k}\right) \hat{\lambda }\left( x_{h}\right) }. \end{aligned}$$

where: $D=\frac{1}{|W|} {\sum }_{h} 1/\hat{\lambda }\left( x_{h}\right)$; *r* is the distance at which the function is evaluated; $\hat{\lambda }(x)$ is the estimated intensity from the model at location *x*; $I\left\{ || x_{k}-x_{h}||\right\}$ is an indicator function that takes the value 1 if the absolute distance between any two locations $x_{k}$ and $x_{h}$ is less or equal to *r*, and 0 otherwise.

We then validate our model using the following bootstrap procedure.

1. By plugging in the maximum likelihood estimate for $\theta$, simulate a data set based on the inhomogenous marked point process defined in the previous section.
2. Compute the inhomogeneous K-function defined in (5) for the simulated data set in the previous step.
3. Repeat steps (1) and (2) 10,000 times.
4. For a set of predefined distances *r* compute the 95$\%$ confidence intervals using the 10,000 functions obtained from the previous steps.

On completion of the last step, we then conclude that the data do not show evidence against the fitted model if the K-function computed on the original data falls within the 95$\%$ envelope for each of the age-gender combinations.

### Ethics consideration

The Oxford Tropical Research Ethics Committee (reference number 39-15) and the Malawian National Health Sciences Research Committee (reference number 15/5/1599) gave the approval to conduct the STRATAA study (trial number ISRCTN 12131979) in Malawi^6^. At the household level, the head of the household provided written informed consent for household surveys on behalf of the entire household. In the other components of the STRATAA study, an informed consent form was signed by study participants aged at least 18 years. On the other hand, informed consent forms were signed by parents or guardians of children less than 18 years old. Assent was, additionally, sought from children aged between 11 and 17 years. We confirm that the methods performed in this study were conducted in accordance with appropriate regulations and guidelines. Furthermore, we confirm that the study complies with the Declaration of Helsinki.

## Results

**Figure 1:**
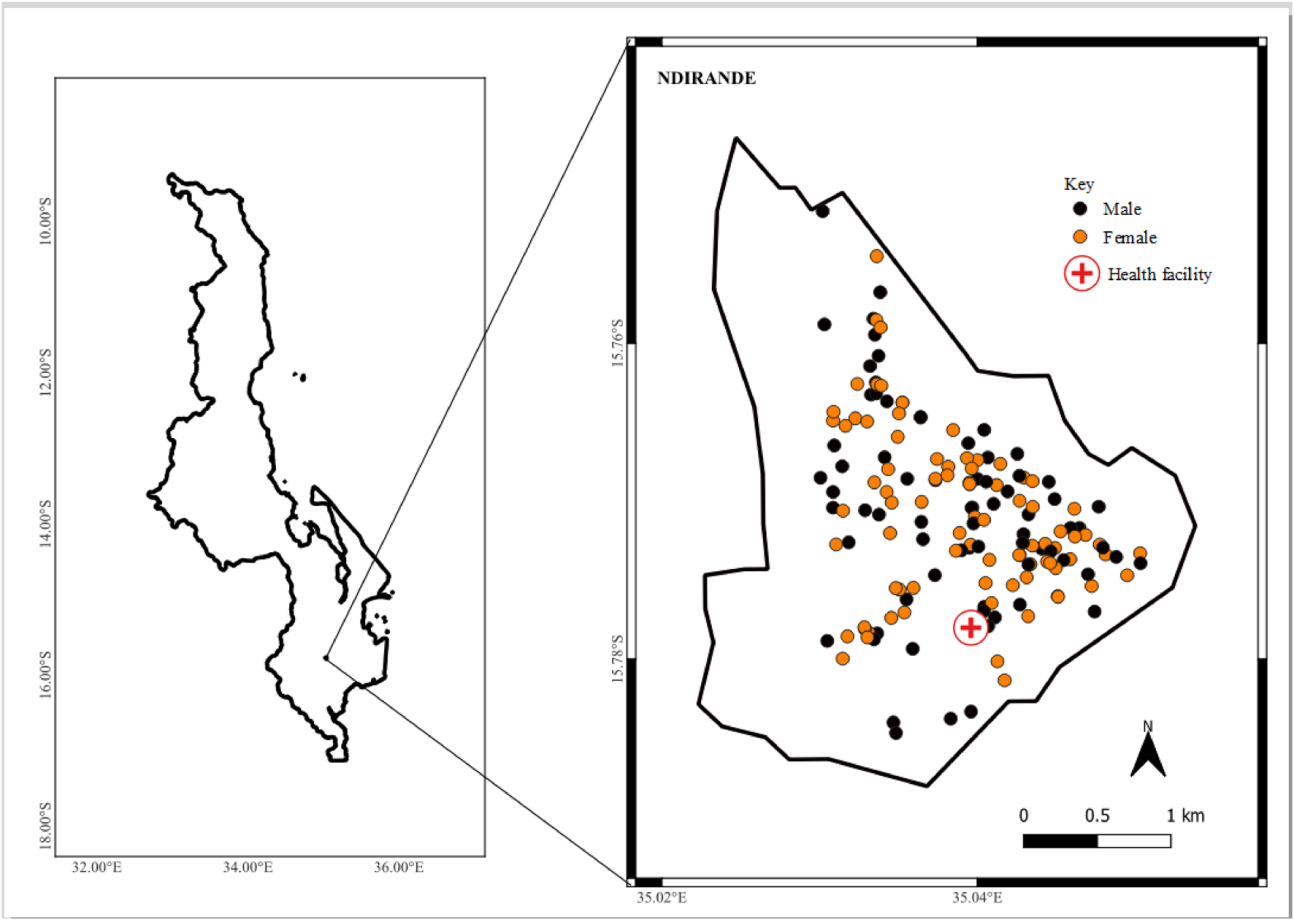
Locations of 160 typhoid cases and Ndirande health clinic from October 2016 to February 2020. The shaded area represents the study region.

A total of 161 typhoid cases were recorded at Ndirande Health clinic between October 2016 and February 2020. Out of these, only 1 case did not have complete information on age, gender and the date of sample collection. The analysis presented is thus based on the 160 typhoid cases with no missing data. A total of 43% (n = 69) of the study participants were aged between 6 and 17 years. The median age of the study participants was 11 years (interquartile range, IQR: 6–21 years). Further, 52% (n = 83) of the sample were females. Figure 1 shows the distribution of typhoid cases by gender in Ndirande. Table 1 further summarises the characteristics of the sample.

**Table 1:** Distribution of the study participants.

Variable	Total (n)	Percentage (%)
Age (median, IQR)	11 years (6–21 years)	
Age (years)		
0-5	32	20
6-17	69	43
18+	49	37
Gender		
Male	77	48
Female	83	52

**Figure 2:**
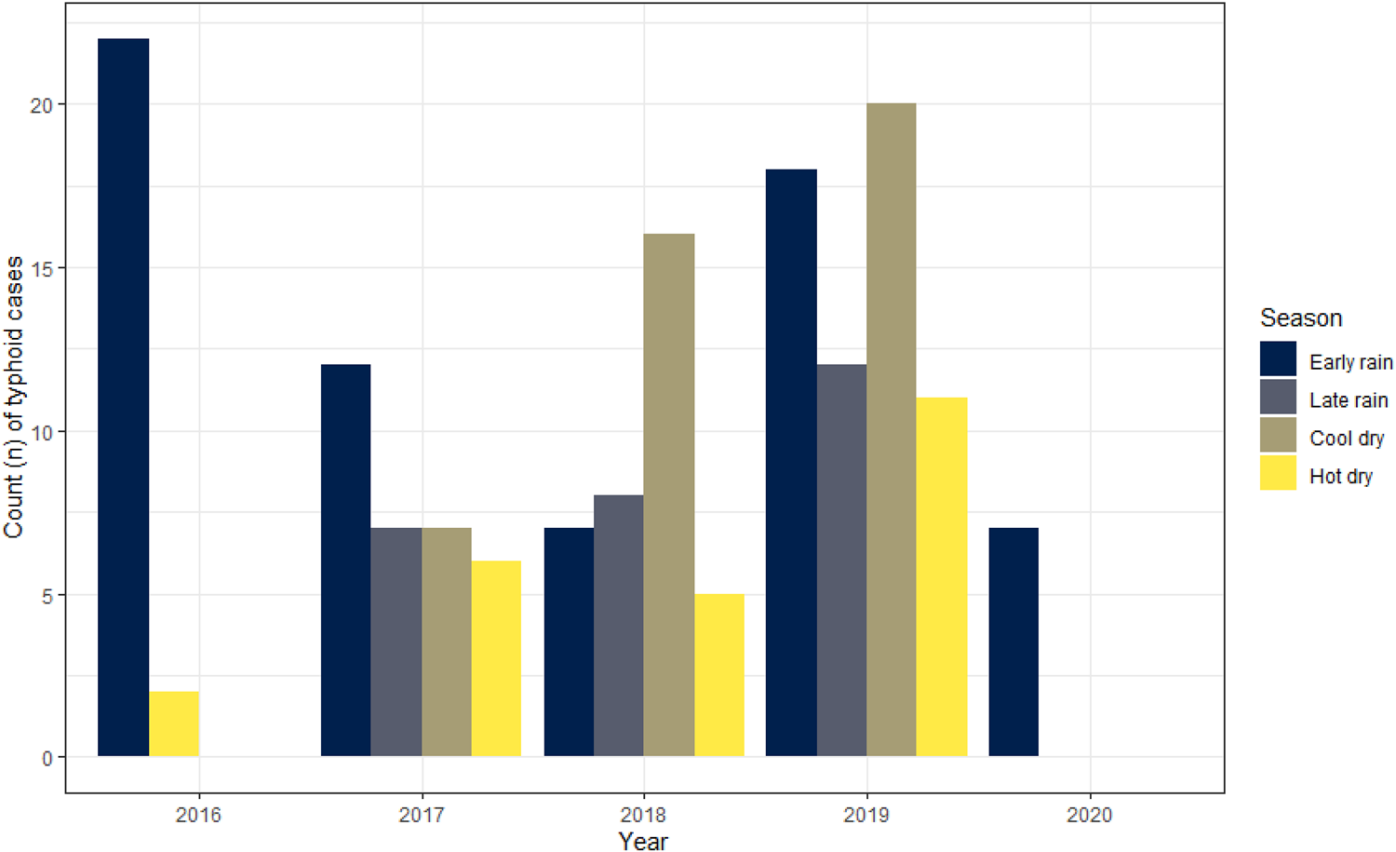
Observed typhoid cases per season from October 2016 to February 2020.

Figure 2 illustrates the typhoid cases recorded per season from October 2016 to February 2020. This plot does not show any discernible temporal pattern.

Our study results show that a 50 meters increase in the distance away from the health clinic decreased the estimated incidence rate of typhoid by 1% (100 * {1 - exponent of coefficient (coef): − 0.01}, 95% confidence interval (CI): − 0.03, 0.01). Further, a 50 meters increase in the elevation decreased the estimated incidence rate of typhoid by 9% (coef: − 0.10, 95% CI: − 0.42, 0.12). With further regard to the spatial covariates, a one-unit increase in the WASH score was associated with a decrease in the incidence rate of typhoid of 54% (coef: − 0.78, 95% CI: − 1.34, − 0.45). We find that only the WASH score shows a significant effect at the $5\%$ conventional confidence level. However, all the point estimates of the regression component align with the expected direction, as informed by our understanding of typhoid fever epidemiology.

**Table 2:** Maximum likelihood estimates and 95$\%$ confidence intervals (CI) for the parameters of the model specified in (3).

Variable	Estimate	95% CI
Age (years)		
0–5	− 3.119	(− 5.147, − 0.193)
6–17	− 3.162	(− 5.189, − 0.230)
18+	− 3.906	(− 5.929, − 0.973)
Gender		
Male	− 5.140	(− 8.177, − 0.746)
Female	− 5.047	(− 8.087, − 0.652)
Spatial covariates		
Distance to health facility $\times$ 50 meters	− 0.010	(− 0.027, 0.008)
Elevation $\times$ 50 meters	− 0.098	(− 0.420, 0.123)
WASH score	− 0.782	(-1.338, -0.449)

**Figure 3:**
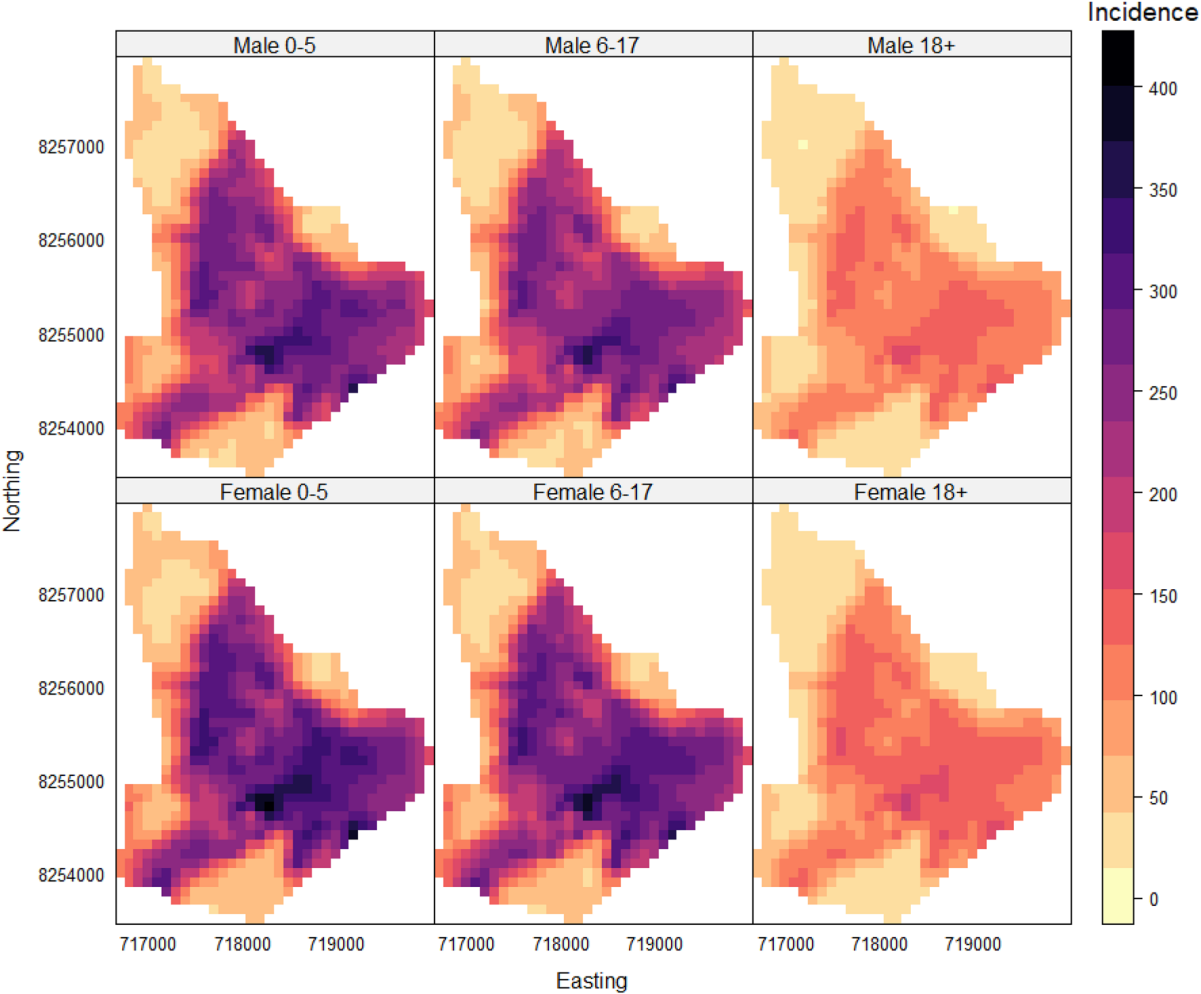
Predicted incidence of typhoid by gender and age per 100,000 population. The rows represent the gender of a typhoid case, whilst the columns represent the age group of the case.

Predicted relative intensities were computed and plotted for each combination of marks (age and gender) while adjusting for the spatial covariates and population. Figure 3 shows the average predicted reported incidence for males and females of any age at any point in time in the study per 100,000 population. As can be seen in Fig. 3, the areas with the highest typhoid risk were the central and southeast areas of Ndirande. The highest predicted reported incidence overall was in females (400 typhoid cases per 100,000 population) and males (365 typhoid cases per 100,000 population) aged between 0 and 5 years. This finding concurs with the model coefficients reported in Table 2. When comparing the adjusted predicted reported incidences within each gender, the 0–5 age group had the highest predicted relative intensity for both males and females per 100,000 population per month, as shown in Table 3.

**Table 3:** Predicted incidence and 95% confidence intervals (CI) per 100,000 population for Ndirande; for the definition of the predictive target see Eq. 4.

Group	Number	Incidence rate	95% CI
Male 0–5	14	222	(219, 224)
Male 6–17	36	216	( 215, 216)
Male 18+	27	104	(103, 105)
Female 0–5	18	240	(238, 242)
Female 6–17	33	237	(236, 237)
Female 18+	32	114	(113, 115)

We fitted an inhomogeneous K-function to validate our spatial point pattern model. The model validation plots for the final model are attached in the supplementary material. Overall, the figures show that the K-functions from the observed data mostly fell within the simulated envelope for most of the distances. This suggests that our model was a good fit for the data.

## Discussion

In this study, we have shown how spatial point pattern methods can be used to analyze reported cases of typhoid fever in health facilities. Our approach based on a multiple-marked inhomogeneous Poisson process model allowed us to estimate typhoid incidence at the household level while adjusting for both spatial and individual-level risk factors.

Several modelling challenges were encountered in the analysis. First, the small number of reported cases over time and space makes understanding the relationships between risk factors and the overall incidence patterns more difficult to model. In this context, the interpretation of the regression relationships should not only be guided by statistical summaries, such as *p*-values, but prior knowledge about the disease context should also be used to inform the selection of covariates. For this reason, we decided to retain variables that were not statistically significant, namely distance to a health facility, and elevation, to generate the spatial predictions for typhoid fever incidence. Our general guiding principle is that a variable should be retained in the final model, regardless of its statistical significance, (1) if there is an established body of evidence on the importance of the variable to model the health outcome of interest, and (2) if the point estimate is in accordance with the expected direction of the relationship based on that prior knowledge. In the case of the three variables considered, it has been established in previous research that these three variables are important risk factors for typhoid^4,5,22,23^ and both of the aforementioned criteria are met.

Based on the effects of these risk factors, the southeast zone of Ndirande was found to show the highest typhoid incidence rate. This area of Ndirande is characterized by a high population density which could contribute to poor sanitary facilities as indicated by the poor WASH facilities. Our incidence map provides a more granular distribution of typhoid compared to previous work^18^. The finding on typhoid incidence decreasing with good WASH facilities is in line with the findings from another study carried out in Blantyre district in Malawi^5^. The result of an increase in the elevation being associated with a decrease in the incidence of typhoid is also consistent with results from previous studies^4,23^. The maximum distance observed between the health center and the study area was recorded as 3.1 km. Our results further showed that an increase in the distance to the Ndirande health clinic was associated with a decrease in the reported incidence of typhoid. This suggests that people living far away may be more reluctant to go to the clinic unless they are seriously ill^22^. It is important to note, however, a potential limitation of these findings. The GPS coordinates used in this study were collected at the household level, and thus may not reflect the true locations of the exposure to typhoid.

In addition to the spatial (environmental) risk factors, the age of an individual is found to play an important role in the variation of typhoid risk. Our study findings indicate a higher occurrence of typhoid among children after adjusting for the spatial covariates. This result is consistent with previous studies that also reported a higher typhoid incidence among children compared to adults^2,14,18,29^. The estimated typhoid intensities for the 3 age groups in this study are, however, lower than the adjusted typhoid incidences recently reported in Blantyre in Malawi because we did not adjust the incidence in our study by a number of factors such as blood culture sensitivity and healthcare-seeking probability^12,30^. In contrast to previous studies, we did not find any statistically significant difference in the estimated incidence between females and males^31,32^.

Another important limitation of this study is the under-reporting arising from passive surveillance data collected from individuals who visit a health facility^1,33,34^. To account for the under-reporting, our model can be extended in future work using a thinned inhomogeneous Poisson process model, whereby the intensity of the Poisson process is scaled by the probability of visiting the health centre^28^. However, one of the challenges of this approach is that some covariates may affect both typhoid fever risk and the probability of visiting a clinic, making the estimation of regression relationships more problematic. This issue has also been reported in ecology, where similar methods have been used in citizen science data^35^. Future research should focus on a better understanding of the factors and mechanisms that drive the likelihood of attending health facilities, to better parameterise the probability of going to the hospital and overcome the identifiability issues in the estimation.

The proposed modelling approach in this study may be applied to the analysis of reported cases from passive surveillance data for other diseases. One of the strengths of the illustrated modelling approach is its flexibility in being adapted to any other environmentally driven diseases through the selection of suitable covariates. Through the application of this approach, we have further demonstrated that, for example, typhoid occurrence is higher among children and in areas with households with poor WASH facilities. Optimal typhoid control initiatives could focus on this age group and on improving WASH facilities in households.

### Supplementary Information

Supplementary Information.

## Data Availability

The data that support the findings of this study are available from the chief investigator, Professor Andrew Pollard, but restrictions apply to the availability of these data, which were used under license for the current study, and so are not publicly available. Data are, however, available from the corresponding author upon reasonable request and with permission of the chief investigator (andrew.pollard@paediatrics.ox.ac.uk). The code used to run the models in this study can be accessed on *Github*.

## References

[1] Stanaway, J. D. *et al.* The global burden of typhoid and paratyphoid fevers: A systematic analysis for the global burden of disease study 2017. *Lancet Infect. Dis.* **19**, 369–381 (2019). 30792131 10.1016/S1473-3099(18)30685-6 PMC6437314

[2] Antillón, M. *et al.* The burden of typhoid fever in low-and middle-income countries: A meta-regression approach. *PLoS Neglect. Trop. Dis.* **11**, e0005376 (2017). 10.1371/journal.pntd.0005376 PMC5344533 28241011

[3] Saad, N. J. *et al.* Seasonal dynamics of typhoid and paratyphoid fever. *Sci. Rep.* **8**, 1–9 (2018). 29720736 10.1038/s41598-018-25234-w PMC5932015

[4] Akullian, A. *et al.* Environmental transmission of typhoid fever in an urban slum. *PLoS Neglect. Trop. Dis.* **9**, e0004212 (2015). 10.1371/journal.pntd.0004212 PMC4669139 26633656

[5] Gauld, J. S. *et al.* Domestic river water use and risk of typhoid fever: Results from a case-control study in Blantyre, Malawi. *Clin. Infect. Dis.* **70**, 1278–1284 (2020). 31144715 10.1093/cid/ciz405

[6] Darton, T. C. *et al.* The strataa study protocol: A programme to assess the burden of enteric fever in Bangladesh, Malawi and Nepal using prospective population census, passive surveillance, serological studies and healthcare utilisation surveys. *BMJ Open* **7**, e016283 (2017). 28674145 10.1136/bmjopen-2017-016283 PMC5726077

[7] Thindwa, D., Chipeta, M. G., Henrion, M. Y. & Gordon, M. A. Distinct climate influences on the risk of typhoid compared to invasive non-typhoid *Salmonella* disease in Blantyre, Malawi. *Sci. Rep.* **9**, 1–11 (2019). 31889080 10.1038/s41598-019-56688-1 PMC6937328

[8] Breiman, R. F. *et al.* Population-based incidence of typhoid fever in an urban informal settlement and a rural area in Kenya: Implications for typhoid vaccine use in Africa. *PloS One* **7**, e29119 (2012). 22276105 10.1371/journal.pone.0029119 PMC3261857

[9] Fusheini, A. & Gyawu, S. K. Prevalence of typhoid and paratyphoid fever in the Hohoe municipality of the Volta region, Ghana: A 5-year retrospective trend analysis. *Ann. Glob. Health* **86**, 111 (2020). 32944508 10.5334/aogh.2833 PMC7473205

[10] World Health Organization*.**Typhoid and Other Invasive Salmonellosis* 1–13 (Vaccine-preventable diseases surveillance standards, 2018).

[11] Meiring, J. E. *et al.* Typhoid vaccine acceleration consortium Malawi: A phase III, randomized, double-blind, controlled trial of the clinical efficacy of typhoid conjugate vaccine among children in Blantyre, Malawi. *Clin. Infect. Dis.* **68**, S50–S58 (2019). 30845320 10.1093/cid/ciy1103 PMC6405268

[12] Meiring, J. E. *et al.* Burden of enteric fever at three urban sites in Africa and Asia: A multicentre population-based study. *Lancet Glob. Health* **9**, e1688–e1696 (2021). 34798028 10.1016/S2214-109X(21)00370-3 PMC8609278

[13] Musicha, P. *et al.* Trends in antimicrobial resistance in bloodstream infection isolates at a large urban hospital in Malawi (1998–2016): A surveillance study. *Lancet Infect. Dis.* **17**, 1042–1052 (2017). 28818544 10.1016/S1473-3099(17)30394-8 PMC5610140

[14] Feasey, N. A. *et al.* Rapid emergence of multidrug resistant, h58-lineage *Salmonella* typhi in Blantyre, Malawi. *PLoS Neglect. Trop. Dis.* **9**, e0003748 (2015). 10.1371/journal.pntd.0003748 PMC4409211 25909750

[15] Pitzer, V. E. *et al.* Mathematical modeling to assess the drivers of the recent emergence of typhoid fever in Blantyre, Malawi. *Clin. Infect. Dis.* **61**, S251–S258 (2015). 26449939 10.1093/cid/civ710 PMC4596932

[16] Browne, A. J. *et al.* Drug-resistant enteric fever worldwide, 1990–2018: A systematic review and meta-analysis. *BMC Med.* **18**, 1–22 (2020). 31898501 10.1186/s12916-019-1443-1 PMC6941399

[17] Gauld, J. S., Bilima, S., Diggle, P. J., Feasey, N. A. & Read, J. M. Rainfall anomalies and typhoid fever in Blantyre, Malawi. *Epidemiol. Infect.* **150**, e122 (2022). 35535751 10.1017/S0950268822000759 PMC9254155

[18] Gauld, J. S. *et al.* Spatial and genomic data to characterize endemic typhoid transmission. *Clin. Infect. Dis.* **74**, 1993–2000 (2022). 34463736 10.1093/cid/ciab745 PMC9187325

[19] Osei, F. B., Stein, A. & Nyadanu, S. D. Spatial and temporal heterogeneities of district-level typhoid morbidities in Ghana: A requisite insight for informed public health response. *Plos One* **13**, e0208006 (2018). 30496258 10.1371/journal.pone.0208006 PMC6264858

[20] Ismail, K., Maiga, G., Ssebuggwawo, D., Nabende, P. & Mansourian, A. Spatio-temporal trends and distribution patterns of typhoid disease in Uganda from 2012 to 2017. *Geospat. Health*10.4081/gh.2020.860 (2020). 10.4081/gh.2020.860 33461278

[21] Tango, T. Spatial scan statistics can be dangerous. *Stat. Methods Med. Res.* **30**, 75–86 (2021). 33595399 10.1177/0962280220930562

[22] Khan, M. I. *et al.* Risk factors associated with typhoid fever in children aged 2–16 years in Karachi, Pakistan. *Epidemiol. Infect.* **140**, 665–672 (2012). 21676350 10.1017/S0950268811000938

[23] Baker, S. *et al.* Combined high-resolution genotyping and geospatial analysis reveals modes of endemic urban typhoid fever transmission. *Open Biol.* **1**, 110008 (2011). 22645647 10.1098/rsob.110008 PMC3352080

[24] Tatem, A. J. Worldpop, open data for spatial demography. *Sci. Data* **4**, 1–4 (2017). 10.1038/sdata.2017.4 PMC5283060 28140397

[25] Diggle, P. J., Kaimi, I. & Abellana, R. Partial-likelihood analysis of spatio-temporal point-process data. *Biometrics* **66**, 347–354 (2010). 19673863 10.1111/j.1541-0420.2009.01304.x

[26] Berman, M. & Turner, T. R. Approximating point process likelihoods with glim. *J. R. Stat. Soc.: Ser. C (Appl. Stat.)* **41**, 31–38 (1992).

[27] Efron, B. *The Jackknife, the Bootstrap and Other Resampling Plans* (SIAM, 1982).

[28] Baddeley, A., Rubak, E. & Turner, R. *Spatial Point Patterns: Methodology and Applications with R* (CRC Press, 2015).

[29] Crump, J. A., Luby, S. P. & Mintz, E. D. The global burden of typhoid fever. *Bull. World Health Organ.* **82**, 346–353 (2004). 15298225 PMC2622843

[30] Phillips, M. T. *et al.* A bayesian approach for estimating typhoid fever incidence from large-scale facility-based passive surveillance data. *Stat. Med.* **40**, 5853–5870 (2021). 34428309 10.1002/sim.9159 PMC9291985

[31] Sattar, A. A. *et al.* Age and gender difference of typhoid fever among paediatric patients attended at a tertiary care hospital in Bangladesh. *Bangladesh J. Infect. Dis.* **3**, 36–39 (2016). 10.3329/bjid.v3i2.33830

[32] Dewan, A. M., Corner, R., Hashizume, M. & Ongee, E. T. Typhoid fever and its association with environmental factors in the Dhaka metropolitan area of Bangladesh: A spatial and time-series approach. *PLoS Neglect. Trop. Dis.* **7**, e1998 (2013). 10.1371/journal.pntd.0001998 PMC3554574 23359825

[33] World Health Organization*.**A Toolkit for National Dengue Burden Estimation* (World Health Organization, Tech. Rep., 2018).

[34] Li, X. *et al.* A spatial hierarchical model for integrating and bias-correcting data from passive and active disease surveillance systems. *Spat. Spatio-Temp. Epidemiol.* **35**, 100341 (2020). 10.1016/j.sste.2020.100341 PMC7704115 33138957

[35] Dissanayake, R. B., Giorgi, E., Stevenson, M., Allavena, R. & Henning, J. Estimating koala density from incidental koala sightings in south-east Queensland, Australia (1997–2013), using a self-exciting spatio-temporal point process model. *Ecol. Evolut.* **11**, 13805–13814 (2021). 10.1002/ece3.8082 PMC8525080 34707819

