# Supplementary material for: Modelling Salmonella Typhi in high-density urban Blantyre neighbourhood, Malawi, using point pattern methods
Source: Sci Rep. 2024 Jul 26;14:17164. doi: 10.1038/s41598-024-66436-9 (PMC11282181; doi:10.1038/s41598-024-66436-9)
Supplement: Supplementary file 1 — Supplementary Information. [file 41598_2024_66436_MOESM1_ESM.pdf]

Supplementary information for the Modelling *Salmonella* Typhi in high-density urban Blantyre neighbourhood, Malawi, using point pattern methods paper

1 Spatial covariates

1.1 Elevation

The elevation raster was downloaded from the Worldpop website [1]. Figure 1 illustrates the elevation in meters in Ndirande township.

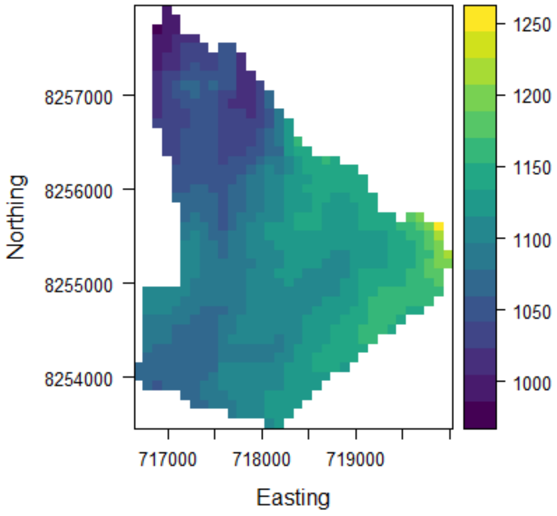

Figure 1: Elevation (meters)

1.2 Distance to the health facility

We calculated the Euclidean distance from each location in Ndirande township to Ndirande health facility. Ndirande health facility is the largest government owned health facility in Ndirande township. The health facility is illustrated as a white star in Figure 2

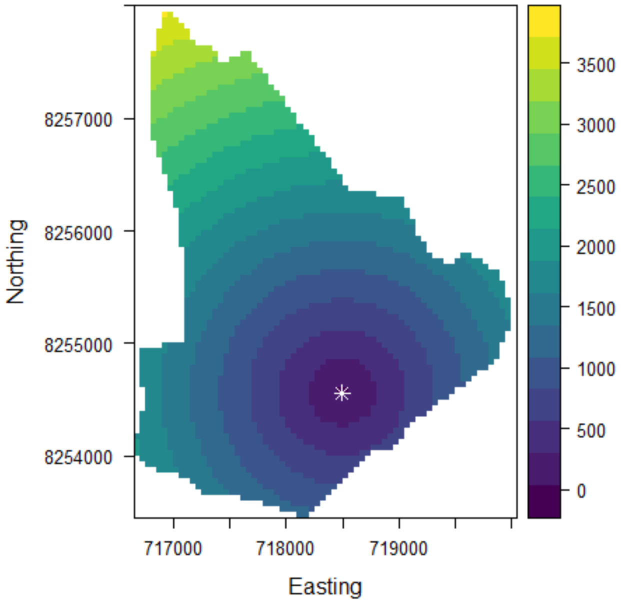

Figure 2: Distance in meters from every location on the grid to Ndirande Health facility.

1.3 Water, sanitation and hygiene (WASH) score

A water, sanitation, and hygiene (WASH) study was carried out in Ndirande township in 2018 as part of the STRATAA study. A total of 14,136 households were sampled in the study. Households were asked several questions related to their WASH and economic levels. Some of the questions asked to these households included:

- The number of rooms a house has (continuous variable).
- The type of toilet used by the house (no toilet facility, toilet shared with other households (public), toilet shared with neighbours and household use only).
- Material of the toilet used by the household (open defecation, pit latrine with a wooden or soil floor, pit latrine with slab, flush or pour toilet).
- The main source of drinking water for a household (borehole and other unprotected sources, public standpipe, piped to the house, protected well or borehole, private tap located outside of the house, public standpipe and public tap outside the house).

A WASH score was derived from the above questions using Principal Components Analysis (PCA). Figure 3 shows the percentage of variation that was explained by the components. It is common practice in epidemiological studies that measure the socioeconomic status of a household to use the first component to derive a desired socioeconomic index or score [2, 3]. Our WASH score was, therefore, based on the first principal component.

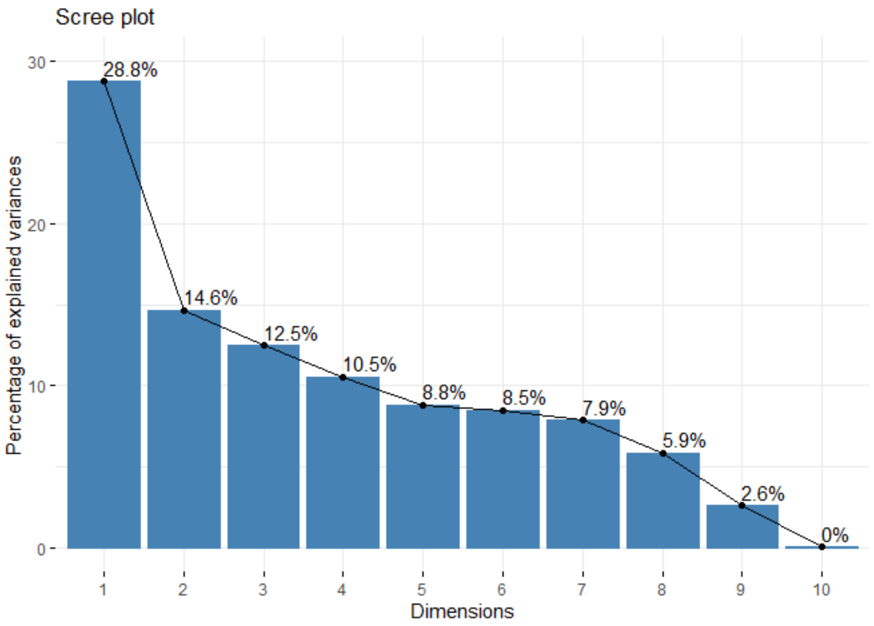

Figure 3: Eigen values illustrating the variance percentage explained by each component

Figure 4 illustrates that the main contributions to the first component of the PCA was from the type of toilet facility and the number of rooms in a house.

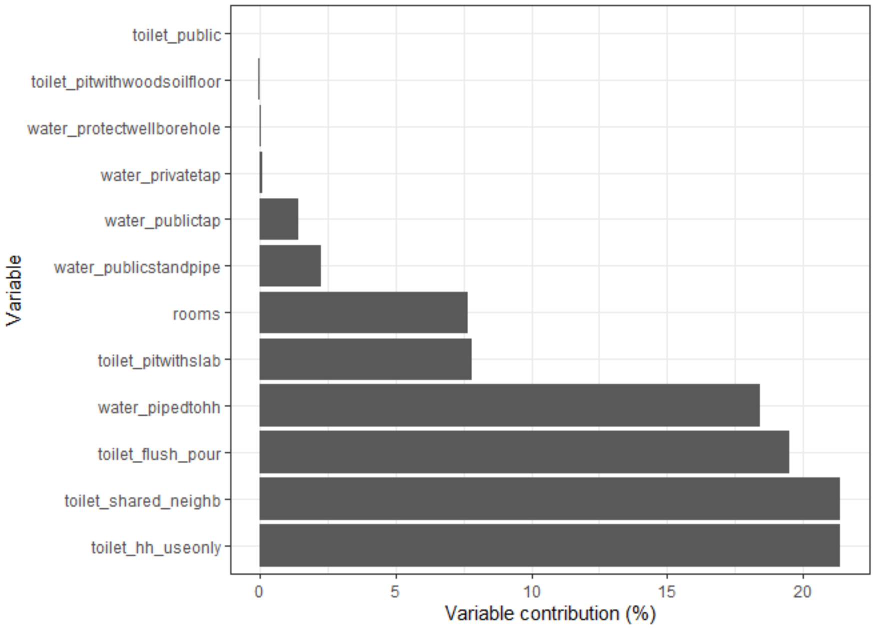

Figure 4: Contribution of variables to the WASH score

The PCA score was then fitted to a linear geostatistical model [4] given below using the PrevMap package [5].

$$Y(x_i) = \mu + Z_i + S(x_i) \tag{1}$$

where $Y(x_i)$ is the observed WASH score at location i, μ is the constant mean effect (intercept), Z_i ($\sim N(0, \tau^2)$) are independently distributed Gaussian variables, and $S(x_i)$

($\sim N(0, \sigma^2)$) is a zero-mean stationary and isotropic Gaussian process.

After assessing the goodness of fit of the model using a semi-variogram, a linear prediction over the whole study area was carried out. This prediction was converted to a raster and used as a covariate in the model.

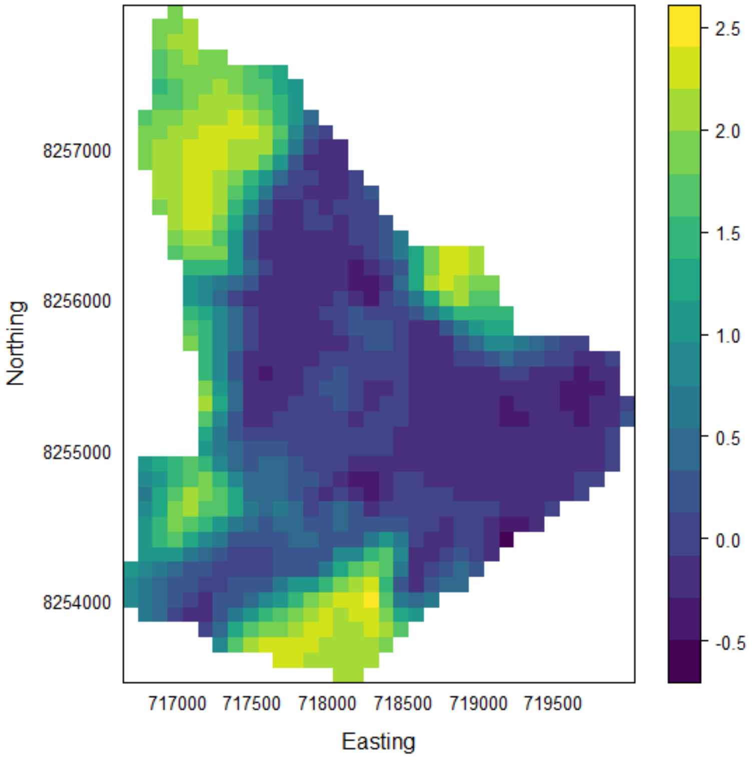

Figure 5: Interpolated Water Sanitation and Hygiene (WASH) score

Figure 6 below illustrates the model validation for the linear geostatistical model used to predict a WASH score throughout Ndirande. The empirical variogram (which shows the residual spatial correlation) is shown in red, whilst the black dotted lines show the simulated envelope for the variogram. Since some parts of the empirical variogram fell outside the simulated envelope, we rejected the null hypothesis of no spatial correlation for the WASH data. We, therefore, concluded that there was some spatial correlation in the WASH survey.

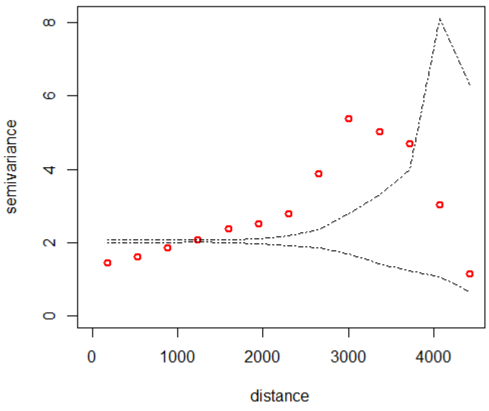

Figure 6: Simulated envelope (black lines) and empirical variogram (red)

2 Ndirande population distribution plots

Figure 7 illustrates the estimated total number of people per grid cell (population count) at 100 m resolution and the estimated population density per grid cell at 1km resolution in Ndirande in 2018. The age-gender specific population distribution plots are presented in Figure 8.

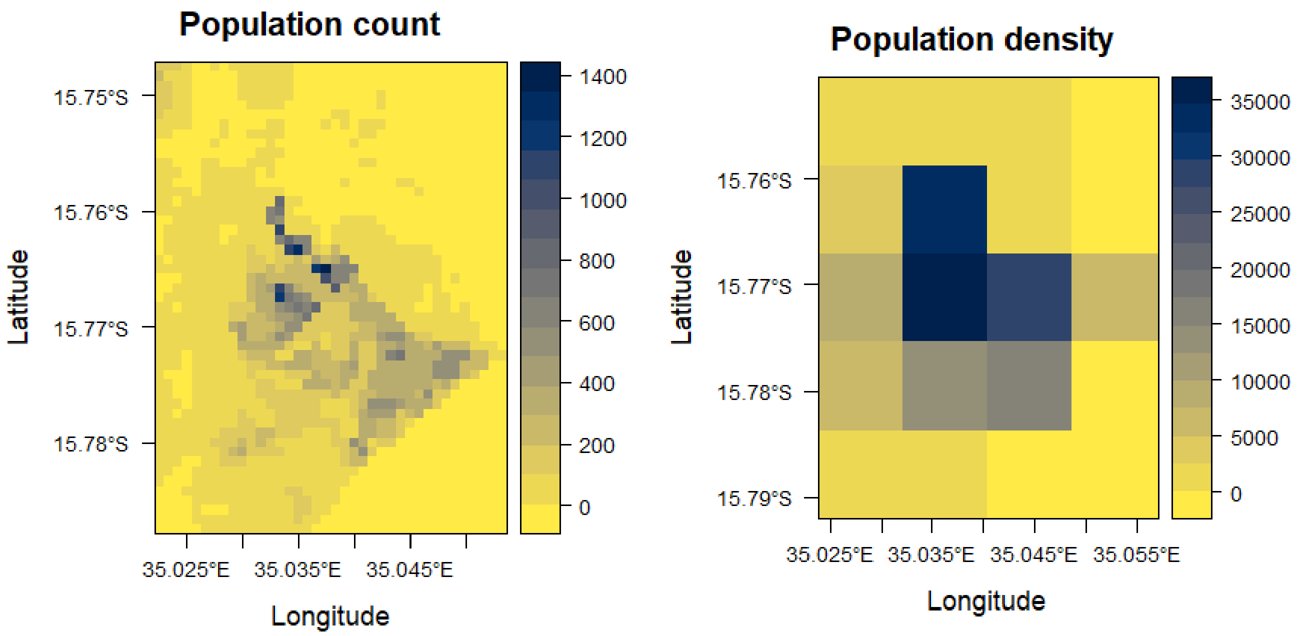

Figure 7: Map of population distribution in Ndirande in 2018.

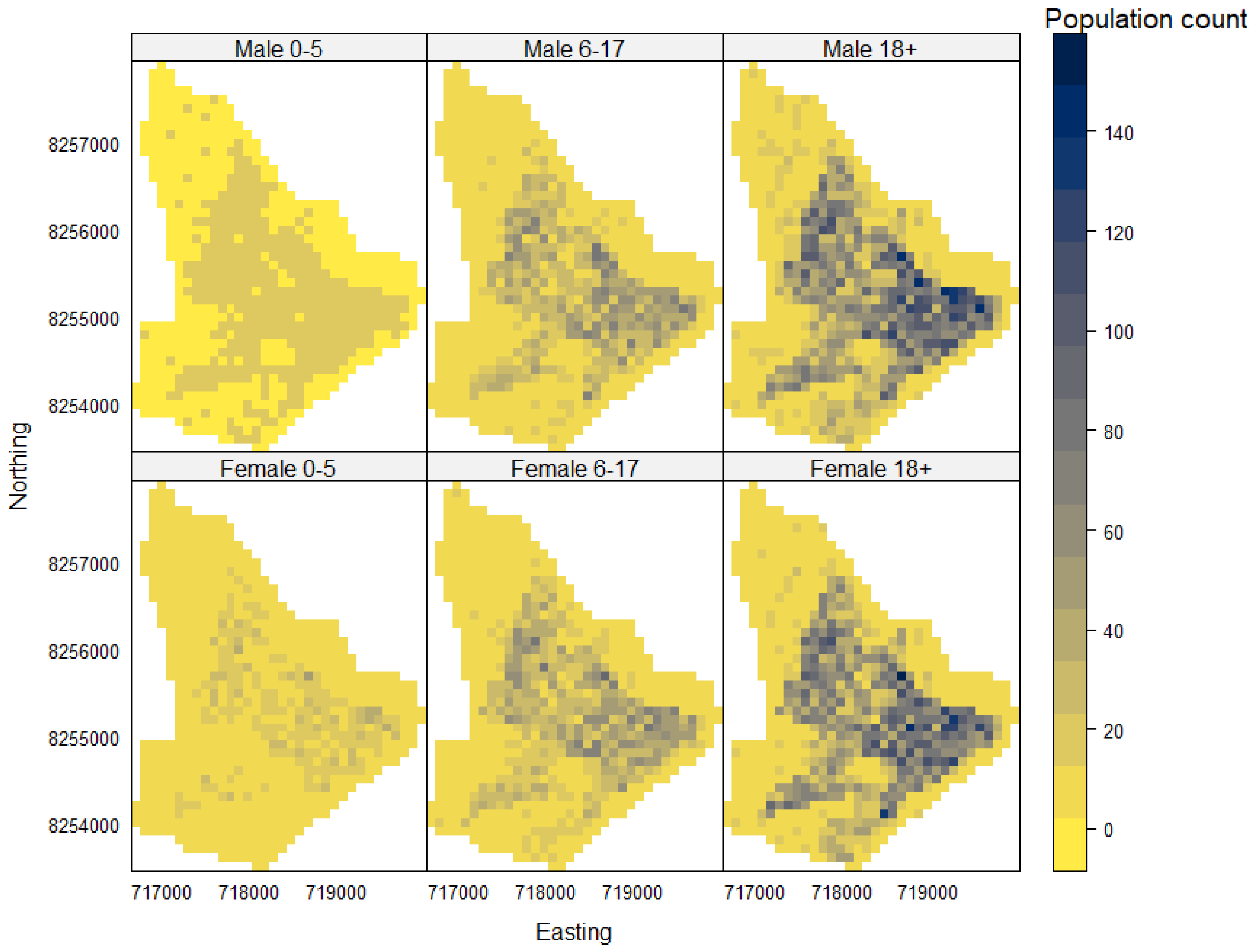

Figure 8: Map of age and gender-specific population distribution in Ndirande in 2018.

3 Model validation plots

We fitted an inhomogeneous K-function to validate our spatial point pattern model. The list of figures below (Figures 9, 10, 11, 12, 13 and 14) show that the K-functions from the observed data mostly fell within the simulated envelope for most of the distances. This suggests that our model was a good fit for the data.

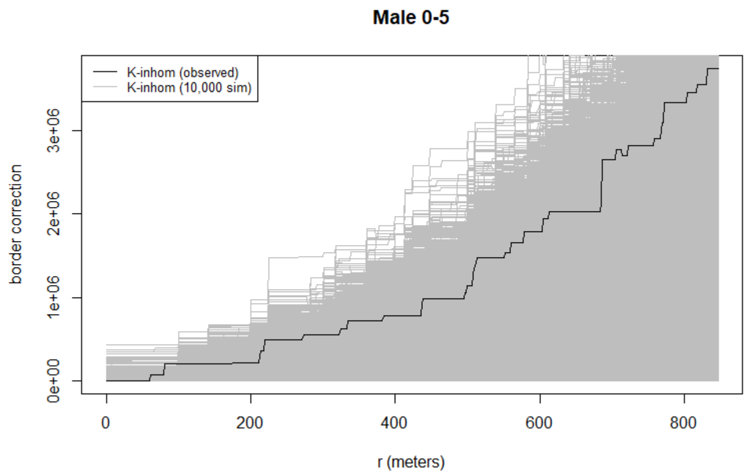

Figure 9: Spatial inhomogeneous K-function for males aged between 0 and 5 years. The black line represents the inhomogeneous K-functions from the observed data, whilst the grey areas represent the inhomogeneous K-functions from the 10,000 realised bootstrap samples

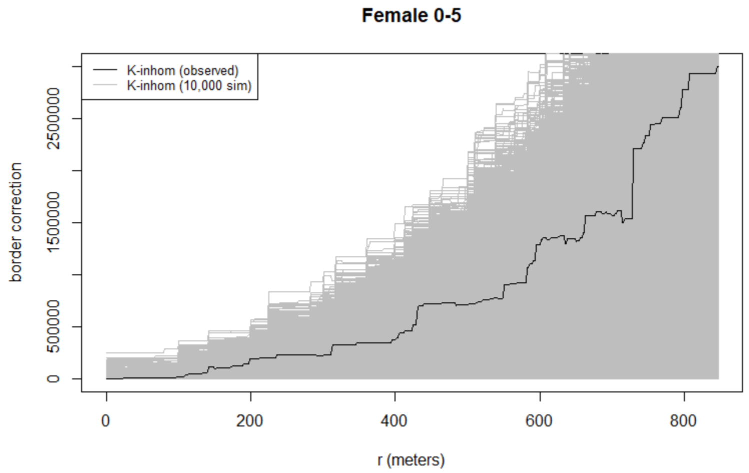

Figure 10: Spatial inhomogeneous K-function for females aged between 0 and 5 years. The black line represents the inhomogeneous K-functions from the observed data, whilst the grey areas represent the inhomogeneous K-functions from the 10,000 realised bootstrap samples

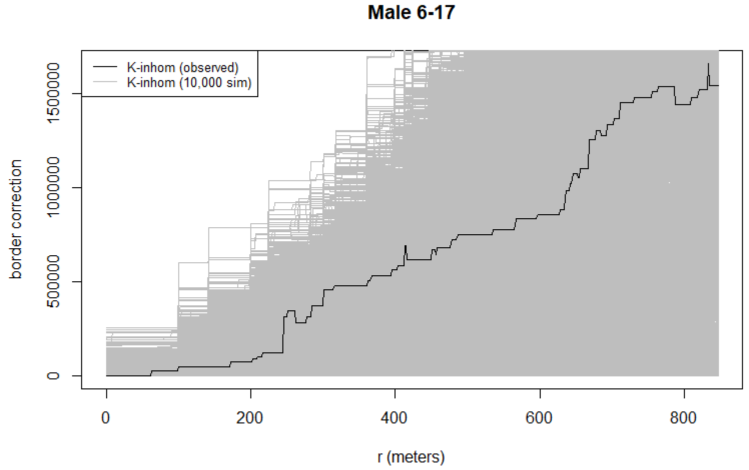

Figure 11: Spatial inhomogeneous K-function for males aged between 6 and 17 years. The black line represents the inhomogeneous K-functions from the observed data, whilst the grey areas represent the inhomogeneous K-functions from the 10,000 realised bootstrap samples

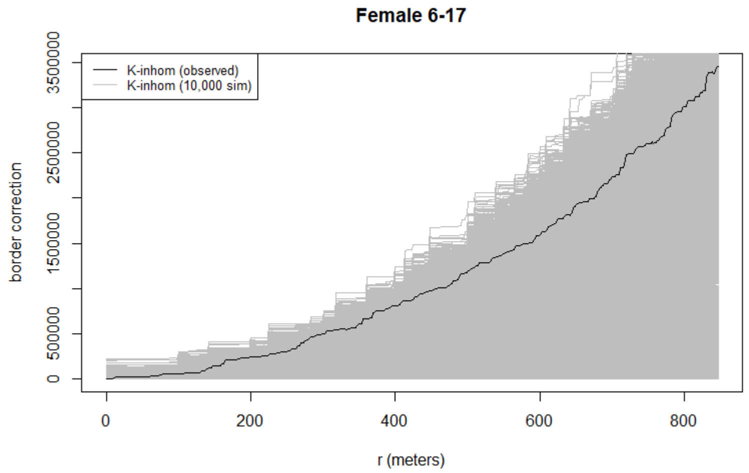

Figure 12: Spatial inhomogeneous K-function for females aged between 6 and 17 years. The black line represents the inhomogeneous K-functions from the observed data, whilst the grey areas represent the inhomogeneous K-functions from the 10,000 realised bootstrap samples

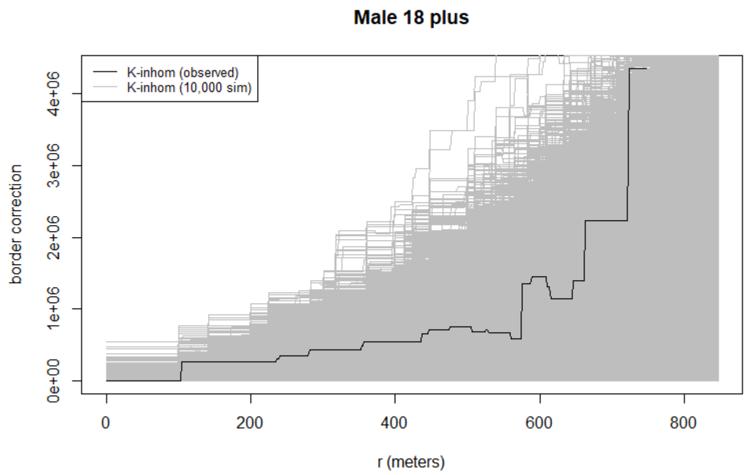

Figure 13: Spatial inhomogeneous K-function for males aged 18 years and above. The black line represents the inhomogeneous K-functions from the observed data, whilst the grey areas represent the inhomogeneous K-functions from the 10,000 realised bootstrap samples

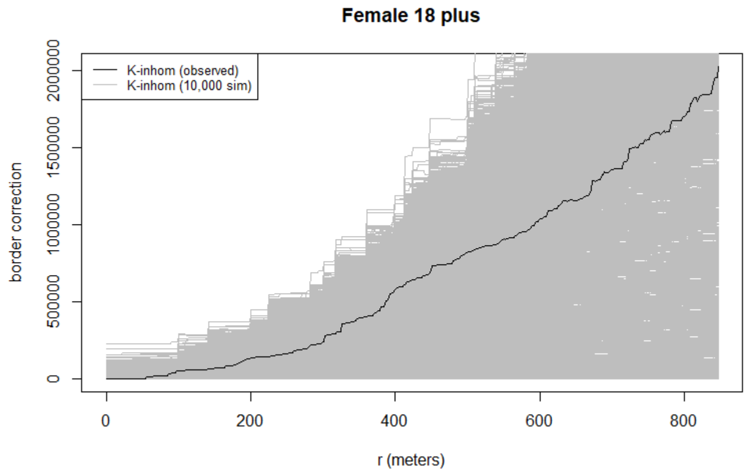

Figure 14: Spatial inhomogeneous K-function for females aged 18 years and above. The black line represents the inhomogeneous K-functions from the observed data, whilst the grey areas represent the inhomogeneous K-functions from the 10,000 realised bootstrap samples

4 Spatio-temporal model

4.1 Model formulation

A spatio-temporal point pattern process can be defined as a realization of a stochastic process whose events are countable [6]. The set of events can be written as (x_k, t_k) where $x_k \in \Re^2$ is the location of an event and $t_k \in \Re^+$ is the time at which event k occurred [7]. The log-likelihood of this process for a marked scenario is given as:

$$L_{ij}(\theta) = \sum_{i=1}^{2} \sum_{j=1}^{3} L_{ij}(\theta) \tag{2}$$

$$L_{ij}(\theta) = \sum_{k=1}^{n_{ij}} \log \lambda_{ij} (x_k, t_k) - \int_A \int_T \lambda_{ij} (x, t) \, dxdt \tag{3}$$

and $\lambda_{ij} (x, t) = \exp \left(\alpha_i + \gamma_j + d (x, t)' \beta + \log m_{ij}(x, t)\right)$ are intensities of the spatial and spatio-temporal point processes. In equation 3:

- x_k for $k = 1, ..., n$ are locations for the observed typhoid cases at time t for a typhoid case with gender i (male or female) and age j (0-5 years, 6-17 years or 18+ years)
- A is the study region and T the temporal region
- $\lambda (x, t)$ is the intensity of the process
- α_i are the intercepts for typhoid case with gender i and γ_j the intercepts for a typhoid case with age j
- $d (x, t)$ is the matrix of spatial and temporal covariates (such as distance to Ndirande health clinic in meters, elevation in meters, WASH score, and season) with their associated coefficients β.
- $m_{ij}(x, t)$ is an offset corresponding to the population for an individual with gender i and age j at location x and time t.

Model 3 uses the same bootstrap procedure for confidence intervals that was defined in the main paper for the purely spatial model.

4.1.1 Model validation

Similar to the purely spatial model defined in the main paper, the spatio-temporal model can be validated using a spatio-temporal inhomogeneous K-function. The inhomogeneous spatio-temporal K-function is given as:

The space-time inhomogeneous function is defined as [8] :

$$K_{AT}(u, v) = 2\pi \int_0^v \int_0^u g (u', v') \, u' du' dv' \tag{4}$$

where

- u is the change in space ($\|x - x'\|$) and v the change in time ($|t - t'|$)
- (u, v) is a vector representing differences in the spatio-temporal domain
- $g(u, v) = \frac{\lambda_2(u,v)}{\lambda(x,t)\lambda(x',t')}$

A non-parametric version of equation 4 can be implemented in the *stpp* software. The non-parametric spatio-temporal inhomogeneous K-function for an infectious disease such as typhoid is mathematically defined as follows [7]:

$$\widehat{K}_{AT}(u,v) = \frac{1}{|A \times T|}\frac{n}{n_v}\sum_{k=1}^{n_v}\sum_{h=1;h>k}^{n_v}\frac{1}{w_{kh}}\frac{1}{\lambda\left(x_k,t_k\right)\lambda\left(x_h,t_h\right)}\mathbf{1}_{\{\|x_k-x_h\|\leq u;t_h-t_k\leq v\}} \quad (5)$$

The parameter w_{kh} in equation 5 denotes the spatial edge correction factor whilst n_v denotes the number of (typhoid) occurrences for which $t_k \leq T_1 - v, T = [T_0, T_1]$ [7].

References

[1] Andrew J Tatem. Worldpop, open data for spatial demography. *Scientific data*, 4(1): 1–4, 2017.

[2] Laura D Howe, Bruna Galobardes, Alicia Matijasevich, David Gordon, Deborah Johnston, Obinna Onwujekwe, Rita Patel, Elizabeth A Webb, Debbie A Lawlor, and James R Hargreaves. Measuring socio-economic position for epidemiological studies in low-and middle-income countries: a methods of measurement in epidemiology paper. *International journal of epidemiology*, 41(3):871–886, 2012.

[3] Lisa Hjelm, Astrid Mathiassen, Darryl Miller, and Amit Wadhwa. Creation of a wealth index. *United Nations World Food Programme*, 2017.

[4] Peter J Diggle and Emanuele Giorgi. *Model-based geostatistics for global public health: methods and applications.* Chapman and Hall/CRC, 2019.

[5] Emanuele Giorgi and Peter J Diggle. Prevmap: an r package for prevalence mapping. *Journal of Statistical Software*, 78:1–29, 2017.

[6] Peter J Diggle, Irene Kaimi, and Rosa Abellana. Partial-likelihood analysis of spatio-temporal point-process data. *Biometrics*, 66(2):347–354, 2010.

[7] Edith Gabriel, Barry S Rowlingson, and Peter J Diggle. stpp: an r package for plotting, simulating and analyzing spatio-temporal point patterns. *Journal of Statistical Software*, 53:1–29, 2013.

[8] Edith Gabriel and Peter J Diggle. Second-order analysis of inhomogeneous spatio-temporal point process data. *Statistica Neerlandica*, 63(1):43–51, 2009.
